# Supplementary material for: Proanthocyanidins Restore the Metabolic Diurnal Rhythm of Subcutaneous White Adipose Tissue According to Time-Of-Day Consumption
Source: Nutrients. 2022 May 27;14(11):2246. doi: 10.3390/nu14112246 (PMC9182881; doi:10.3390/nu14112246)
Supplement: Supplementary file 1 [file nutrients-14-02246-s001.zip › Figures S1 & S2.pdf]

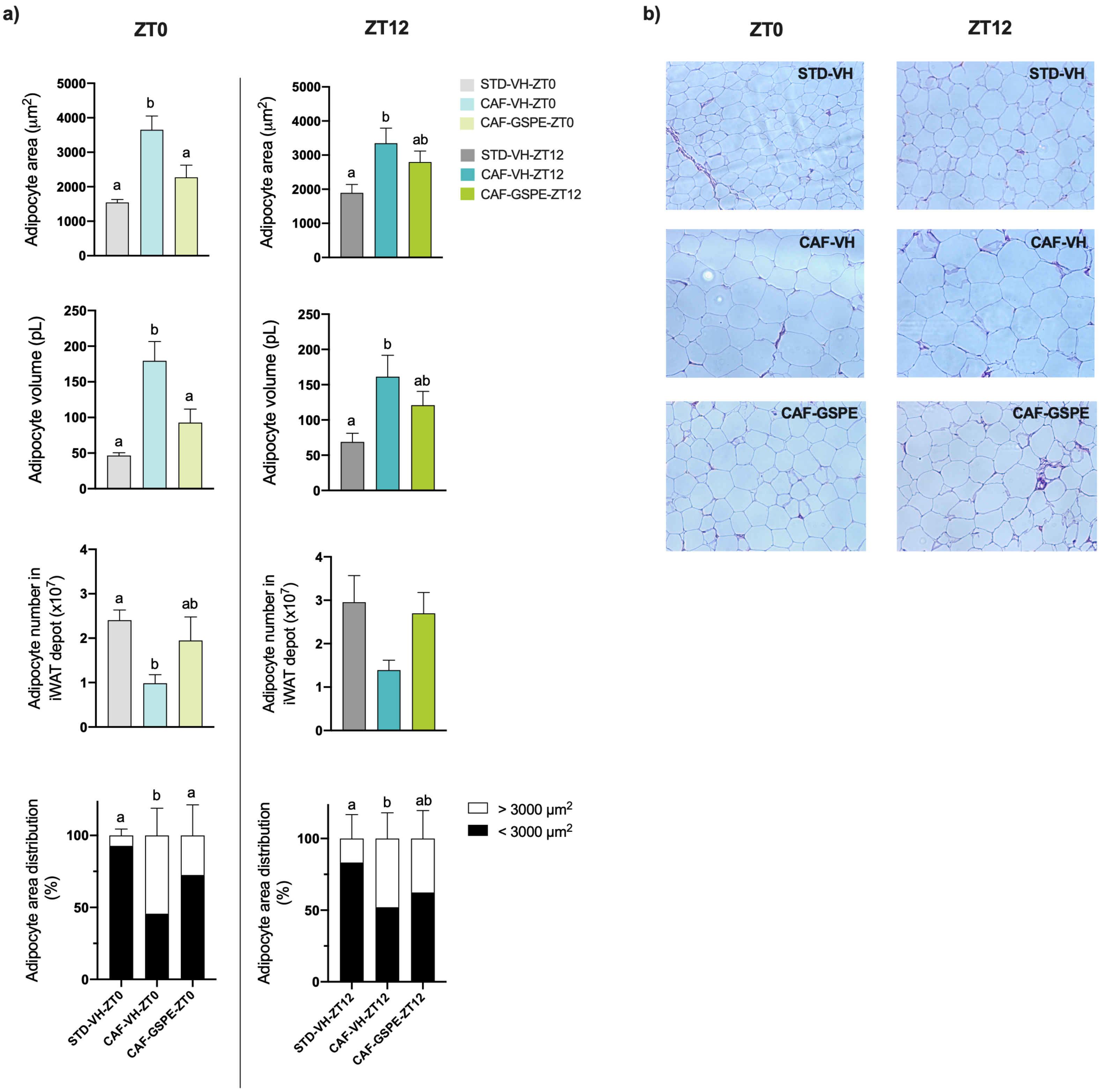

Figure S1. iWAT histology. Histology parameters (a) and representative images of hematoxilin & eosin staining (b). a, b; represent significant differences using one-way ANOVA ($p < 0.05$) followed by Bonferroni posthoc test (n=6, rats sacrificed at ZT1 and ZT7).

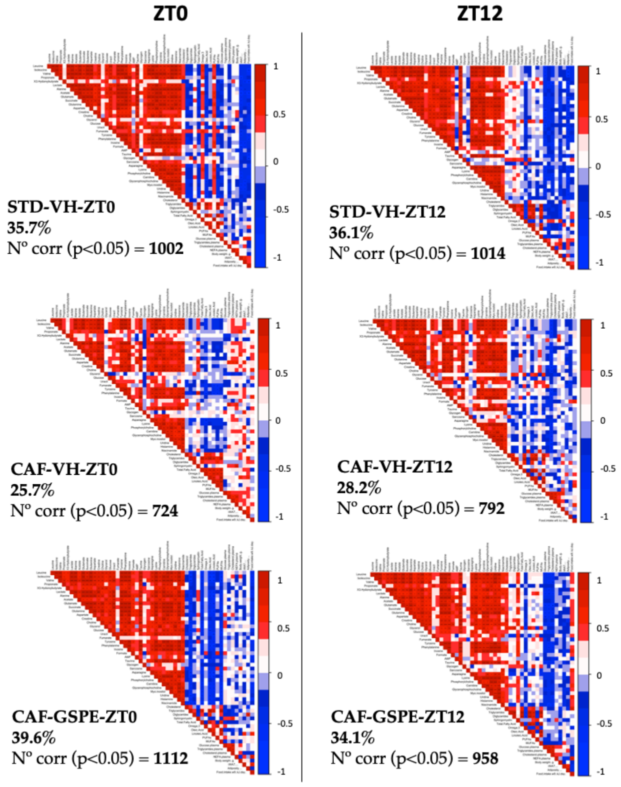

Figure S2. Correlation analysis among iWAT metabolites, final body weight, iWAT relative weight, adiposity and food intake at week 9. * significant correlation analysis using Spearman method ($p < 0.05$). Red and blue mean positive and negative correlations, respectively.
